# Rapidly progressive metastatic cholangiocarcinoma in a postpartum patient with cystic fibrosis: a case report

**DOI:** 10.1186/s12890-020-01337-x

**Published:** 2020-11-16

**Authors:** Sara W. Carson, Kelly E. Craven, David Nauen, Kristina Montemayor, Mark Yarchoan, William R. Burns, Christian A. Merlo, Natalie E. West

**Affiliations:** 1 Department of Medicine, Division of Pulmonary and Critical Care Medicine, Johns Hopkins University, 1830 E. Monument St 5th Floor, Baltimore, MD 21205 USA; 2 Department of Pathology, Johns Hopkins University, Baltimore, MD USA; 3 Department of Oncology, Johns Hopkins University, Baltimore, MD USA; 4 Department of Surgery, Division of Surgical Oncology, Johns Hopkins University, Baltimore, MD USA; 5 Department of Epidemiology, Johns Hopkins Bloomberg School of Public Health, Baltimore, MD USA

**Keywords:** Cholangiocarcinoma, Gastrointestinal cancer, Cystic fibrosis, pregnancy, Case report

## Abstract

**Background:**

Cholangiocarcinoma is a rare gastrointestinal malignancy that arises within the intrahepatic, perihilar, and/or extrahepatic bile ducts. Individuals with cystic fibrosis are at increased risk for gastrointestinal malignancies. The most common gastrointestinal malignancy in cystic fibrosis is colon cancer, but other gastrointestinal malignancies also occur at greater rates than the general population.

**Case presentation:**

We present a case of a rapidly progressive metastatic intrahepatic cholangiocarcinoma in an individual with cystic fibrosis who was 5 months postpartum, incidentally found while undergoing a lung transplantation evaluation.

**Conclusion:**

A heightened clinical awareness of gastrointestinal malignancies, beyond colon cancer, in individuals with cystic fibrosis is warranted. It remains unclear if pregnancy is an additional risk factor for gastrointestinal malignancies in cystic fibrosis.

## Background

Individuals with cystic fibrosis (CF) are at higher risk for digestive tract cancers [1, 2]. Cystic fibrosis transmembrane conductance regulator (CFTR)-regulated chloride channels are highly expressed in the gastrointestinal tract and when disrupted, as in CF, there is increased inflammation and cell turnover, which is believed to contribute to this increased risk [3, 4]. Furthermore, there is limited evidence that pregnancy may decrease the maternal immune system and thus increase the risk of, or at least increase the progression of malignancy [5–8]. However, it is unknown if pregnancy itself would have an additive effect on the increased risk of digestive tract cancers in women with CF. We report a case of a woman with CF who was 5 months postpartum who was diagnosed with rapidly progressive metastatic intrahepatic cholangiocarcinoma incidentally discovered during evaluation for lung transplantation.

## Case presentation

A 26-year-old female with CF (genotype F508del/F508del), with a baseline forced expiratory volume in 1 s (FEV_1_) of 21% predicted and a 6-l home oxygen requirement, was undergoing evaluation for bilateral lung transplantation. Her medical history included recurrent respiratory infections with multi-drug resistant pathogens, pancreatic insufficiency, malnutrition requiring percutaneous gastrostomy feeds, atrioventricular nodal reentry tachycardia status post ablation, and the recent delivery of a healthy baby. She had no history of biliary tract stones, cirrhosis, hepatitis B, or hepatitis C. As part of the lung transplantation workup a CT scan of the chest, abdomen, and pelvis revealed a new peripherally enhancing 6.3 cm mass in the right lobe of her liver, and subsequent MRI suggested intrahepatic cholangiocarcinoma. At this time, the patient reported feeling a hard mass under her right ribs along with intermittent right upper quadrant pain and fullness, anorexia, weight loss, and daily subjective fevers for several weeks.

Ultrasound-guided biopsy identified the mass as an adenocarcinoma, with positive staining for cytokeratin 7 (CK7), GATA-3, a transcription factor important in the regulation of certain genes, and p40, a marker of squamous differentiation. Notably, the tumor was negative for estrogen receptor (ER), thyroid transcription factor 1 (TTF-1), cytokeratin 20 (CK20), and high risk human papillomavirus ribonucleic acid in situ hybridization (HR HPV RNA ISH), and therefore did not support tumor origin of breast or gynecologic, lung, lower gastrointestinal, or cervical or head/neck, respectively. Serum tumor markers demonstrated normal alpha-fetoprotein (AFP) and carcinoembryonic antigen (CEA) levels, but a markedly elevated carbohydrate antigen (CA)19–9 level of 5949.5 U/mL (0–36 U/mL), which can be elevated in pancreatic, gastric, hepatobiliary, and colonic malignancies. Given the tumor location, serum markers, and pathology findings, the tumor was thought to be most consistent with a primary cholangiocarcinoma.

The patient underwent complete staging following hospital discharge, where radiation therapy with curative intent was recommended. Ultimately her poor performance status, chronic pulmonary disease, and risk of severe life-threatening infection influenced the decision not to pursue systemic chemotherapy or surgery as the initial treatment modality. However, prior to starting treatment, she developed pneumonia leading to acute respiratory failure requiring intubation. A second CT of the chest, abdomen, and pelvis (4 weeks from the initial CT) revealed the liver mass had grown to 13.6 × 10 cm and there were patchy pulmonary consolidations concerning for both pneumonia and metastatic disease. Positron emission tomography (PET) scan demonstrated uptake in the lungs bilaterally, mediastinal lymph nodes, right iliac, and right femur, consistent with metastatic disease. Given her critical condition, it was decided she was no longer a candidate for treatment of her cancer. She was transitioned to home hospice where she passed away approximately 10 weeks after the initial CT.

**Fig. 1:**
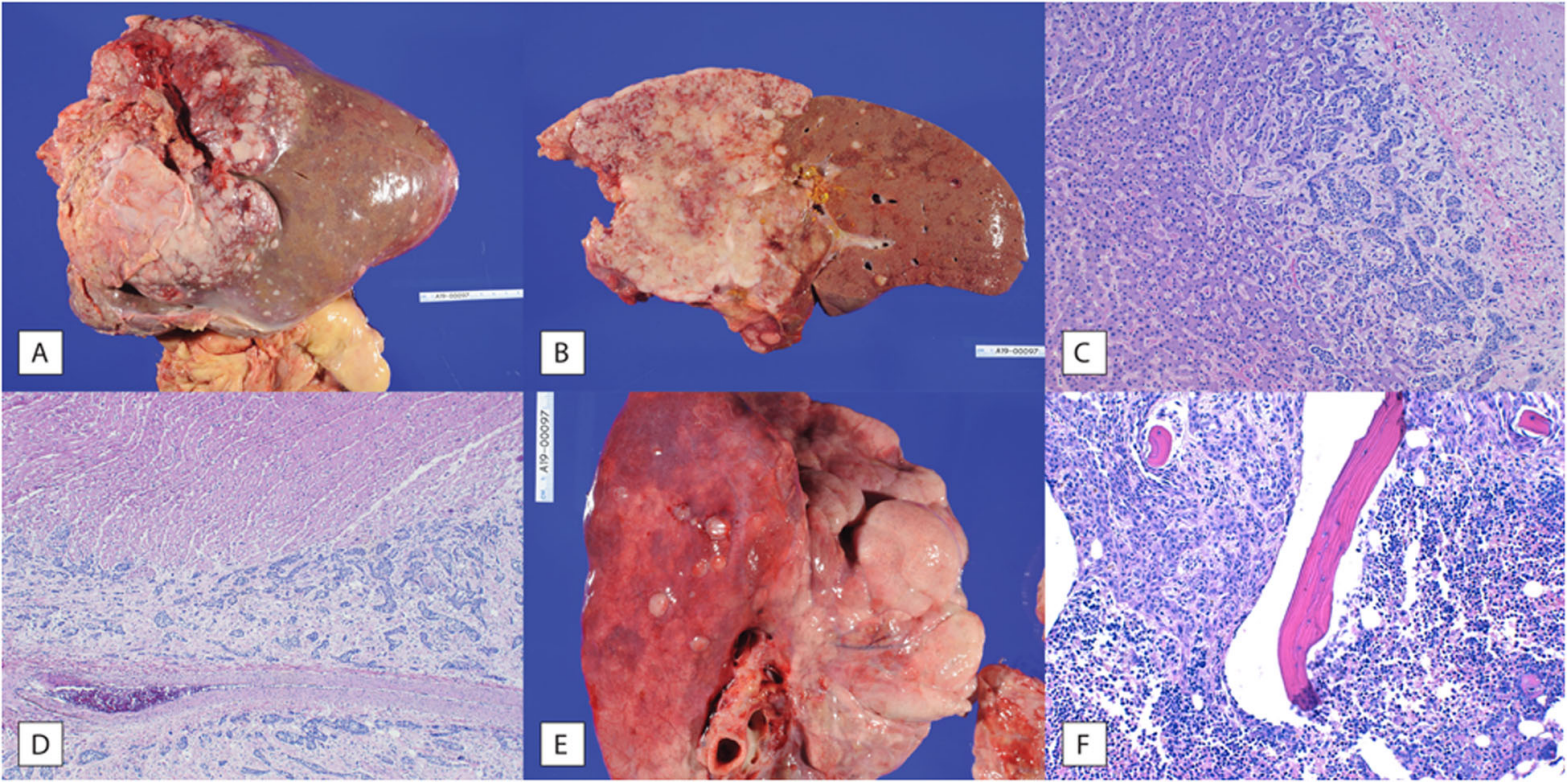
Metastatic Cholangiocarcinoma. **a** Liver with grossly visible tan mass measuring 23 × 20 × 17 cm primarily involving the right lobe and multiple satellite lesions. **b** Cross-section of the liver demonstrating the mass and multiple satellite lesions. **c** A satellite lesion within the liver shows nests of cancerous cells with round to oval nuclei within a fibrotic stroma (right) invading into adjacent normal hepatocyte trabeculae and sinusoids (left). **d** Epicardial and myocardial invasion of metastatic cholangiocarcinoma. Representative section shows nests of tumor cells within a fibrotic stroma around a large vessel (bottom) invading into cardiac myocytes (top). **e** Lung with multiple tan-pink metastatic deposits on the visceral pleura. **f **Bone marrow involvement by metastatic cholangiocarcinoma. Normal trilineage hematopoiesis involving erythroid precursors, myeloid precursors, and megakaryocytes to the right of the bone spicule is replaced by solid/trabecular areas of tumor cells to the left of the bone spicule

Autopsy revealed a 20 × 17 × 23 cm tan mass in the right lobe of the liver (Fig. 1a and b), consistent with intrahepatic cholangiocarcinoma (Fig. 1c), along with metastatic deposits in the heart/epicardium (Fig. 1d), bilateral lungs (Fig. 1e), anterior/posterior cul-de-sacs, left ovary, vaginal adventitia, spleen, diaphragm and bone marrow (Fig. 1f).

Intrahepatic cholangiocarcinoma can resemble adenocarcinomas from other sites, and because there are no histologic markers that can reliably make the distinction, it is often difficult to distinguish them from metastatic tumors [9]. Due to a lack of a dominant tumor at other possible primary sites at autopsy, and a morphology showing anastomosing glands in a fibrotic stroma with focal squamous differentiation, cholangiocarcinoma was favored in this case.

## Discussion

The median life expectancy of individuals with CF has increased significantly over the past few decades, now estimated to be 47.4 years of those born in 2018 [10]. This longer life expectancy has resulted in an increased incidence of some types of cancer among those with CF [1, 2, 11].

Individuals with CF have an increased risk of gastrointestinal cancers, the most common being colon cancer [1, 2]. The first reported case of cholangiocarcinoma in a patient with CF was described by Abdul-Karim in 1982. The patient had a history of CF, recurrent pulmonary infections, pancreatic insufficiency, and biliary tract stones [12]. Since then, to our knowledge, there have been fewer than 10 case reports of biliary tract cancer in those with CF who have not undergone organ transplant [12–14]. In 2003, Maisonneuve and colleagues conducted a 10-year nationwide epidemiologic study that looked at the risk of cancer in non-transplanted and transplanted CF patients and found that both non-transplanted and transplanted CF patients were at greater risk of gastrointestinal cancers, including biliary tract cancers, compared to the age-adjusted population [15]. In 2013, the same authors conducted a 20-year nationwide epidemiologic study to evaluate the risk of cancer in CF and found there was a 3.5-fold increase in all digestive tract cancers compared to the age-adjusted general population. Specifically, the standardized incidence ratios (SIR) for colon cancer was 6.2 (95% CI = 4.2 to 9.0), for small bowel cancer was 11.5 (95% CI 4.2 to 25.4), and for biliary tract cancer was 11.4 (95% CI 3.6 to 27.4) [2]. A recent systematic review and meta-analysis from 2018 reported even greater SIRs for these cancers and found that those with CF who received an organ transplant had a 2–5 times increased risk of developing gastrointestinal cancers compared to those who did not [1].

CFTR-regulated chloride channels, highly expressed in many of the gastrointestinal organs, are known to play an instrumental role in the digestive tract. Therefore, individuals with CF are at greater risk of developing a range of gastrointestinal tract dysfunctions. These include deficient anion (Cl- and HCO3-) and fluid transport, impaired release and clearance of mucus which can lead to meconium ileus and distal intestinal obstructive syndrome (DIOS), pancreatic insufficiency, decreased intestinal lumen pH, increased intestinal stem cell pH, abnormal bacterial colonization, microbial dysbiosis, and impaired innate immune responses that lead to chronic inflammation [3, 16]. As a result, CF individuals have increased chronic intestinal inflammation and intestinal cell turnover which are believed to increase the risk of gastrointestinal cancers among CF individuals [3, 4]. In addition, CFTR is believed to be a tumor suppressor gene and disruption, as in CF, leads to increased risk for colon cancer and other digestive tract cancers [17, 18].

Cholangiocarcinoma refers to cancers of the bile duct that arise in the intrahepatic (< 10% of cases), perihilar, or distal (extrahepatic) biliary tree [19]. Cholangiocarcinoma is rare, with a reported incidence of 2 cases per 100,000 in the US and represents approximately 3% of all gastrointestinal malignancies [20]. Risk factors include chronic biliary tract inflammation due to primary sclerosing cholangitis, choledochal cysts, chronic cholelithiasis/choledocholithiasis, hepatolithiasis, chronic viral and non-viral liver disease, infections such as HIV, *Helicobacter pylori*, and certain parasitic infections, obesity, medications, CF, and several other genetic conditions such as Lynch syndrome, multiple biliary papillomatosis, and BAP1 tumor predisposition syndrome [1, 13, 21, 22]. These risk factors all lead to chronic inflammation and/or cholestasis in the bile duct, which change the microenvironment in the bile duct, increasing the risk of cholangiocarcinoma [23]. In addition, it is likely that there are also successive genetic abnormalities contributing to the pathogenesis involving both oncogenes (RAS, ERBB2, BRAF, EGFR, PIK3CA, CTNNB1) and tumor suppressor genes (e.g. p53, SMAD4, CDKN2A) [24–26].

Other than CF, our patient did not have any known risk factors for cholangiocarcinoma that have been previously described in the literature. However, she was 5 months postpartum at the time of her cancer diagnosis. Although we cannot be certain as to whether the cholangiocarcinoma was present before her pregnancy, this is of particular interest as Qasrawi and colleagues reported a case of cholangiocarcinoma discovered during pregnancy and compared their case to 9 other similar cases in the literature where cholangiocarcinoma was diagnosed during pregnancy [5]. AFP and placental steroids may be responsible for immune system suppression during pregnancy [6, 7]. Furthermore, more aggressive neoplasia in pregnant women is thought to be correlated to the suppression of maternal immunity. The fetus may act as a natural allograft and the nonrejection mechanism that operates in malignancy may also be triggered in pregnancy, making a tumor or a fetus acceptable to the host and may contribute to development or progression of malignancy in susceptible individuals [8]. Data for this proposed mechanism are limited; however, and this theory remains mainly speculative. Although the association between our patient with CF who was pregnant and subsequently was diagnosed with cholangiocarcinoma is of scientific interest, there are only a limited number of reported cases of cholangiocarcinoma cases in pregnancy in the literature and none in CF that we know of. It remains unclear if pregnancy has an additive effect on the risk of development and progression of cholangiocarcinoma among those with CF.

In conclusion, our case demonstrates a unique presentation of a rapidly progressing intrahepatic cholangiocarcinoma in an individual with cystic fibrosis who was 5 months postpartum undergoing lung transplantation workup. It remains unclear as to whether the presentation of her advanced disease was due to immunosuppression of pregnancy or delayed diagnosis because of the pregnancy itself. Our case, supported by the literature, suggests that a heightened clinical awareness for the increased risk of gastrointestinal cancers, beyond colon cancer, and in pregnancy, is warranted in CF.

## Data Availability

Not applicable.

## Abbreviations

CF: Cystic fibrosis
FEV_1_: Forced expiratory volume in 1 s
CK7: Cytokeratin 7
ER: Estrogen receptor
TTF-1: Thyroid transcription factor 1
CK20: Cytokeratin 20
HR HPV RNA ISH: High risk human papillomavirus ribonucleic acid in situ hybridization
AFP: Alpha-fetoprotein
CEA: Carcinoembryonic antigen
CA-19-9: Carbohydrate antigen 19–9
PET: Positron emission tomography
SIR: Standardized incidence ratios
CFTR: Cystic fibrosis transmembrane conductance regulator
DIOS: Distal intestinal obstructive syndrome
